# Epidermal growth factor signalling and bone metastasis

**DOI:** 10.1038/sj.bjc.6605490

**Published:** 2009-12-15

**Authors:** X Lu, Y Kang

**Affiliations:** 1 Department of Molecular Biology, Princeton University, Princeton, NJ, USA; 2 Breast Cancer Program, Cancer Institute of New Jersey, New Brunswick, NJ 08903, USA

**Keywords:** EGF, ERBB, bone metastasis, osteoclast, osteoblast, metalloproteinase

## Abstract

Epidermal growth factor (EGF) signalling is well known for its multifaceted functions in development and tissue homoeostasis. The EGF family of ligands and receptors (ERBB family) have also been extensively investigated for their roles in promoting tumourigenesis and metastasis in a variety of cancer types. Recent findings indicate that EGF signalling is an important mediator of bone metastasis in breast, prostate and kidney cancers. The EGF signalling stimulates the growth of bone metastasis directly by increasing tumour cell proliferation and indirectly by engaging bone stromal cell in metastasis-promoting activities. Therefore, molecular targeting of ERBB receptors may benefit patients with bone metastasis and should be evaluated in clinical trials.

Epidermal growth factor (EGF) signalling is initiated by the binding of EGF family members to the extracellular domain of erythroblastic leukemia viral oncogene homologue (ERBB) receptors. The ERBB receptor tyrosine kinase family consists of four members: epidermal growth factor receptor (EGFR)/ERBB1/HER1, ERBB2/HER2, ERBB3/HER3 and ERBB4/HER4 (Hynes and Lane, 2005; Normanno *et al*, 2006). Each member has distinct binding affinities to different ligands that allows the classification of ligands into three groups: in the first group, EGF, transforming growth factor-*α* (TGF-*α*) and amphiregulin (AREG) specifically bind to EGFR; in the second group, betacellulin, heparin-binding EGF (HB-EGF) and epiregulin bind to both EGFR and ERBB4; the third class include neuregulins (NRGs) that bind to either only ERBB4 (NRG3 and NRG4) or both ERBB3 and ERBB4 (NRG1 and NRG2). None of the EGF family ligands binds to ERBB2, because ERBB2 ectodomain is locked in a conformation that block the ligand-binding site. Despite lacking a cognate ligand, ERBB2 can indirectly mediate signalling when forming heterodimers with a ligand-bound ERBB receptor.

On binding, ERBBs form homo- or heterodimers and activate multiple important pathways involving effectors such as rat sarcoma viral oncogene homologue (RAS)/mitogen-activated protein kinase, phosphatidylinositol 3-kinase-AKT, mammalian target of rapamycin, signal transducer and activator of transcription, SRC tyrosine kinase, phospholipase C-*γ*1/protein kinase C (PKC) and p27 (Hynes and Lane, 2005; Wieduwilt and Moasser, 2008). The activation of these pathways has essential functions in many aspects of development and tissue homoeostasis. Aberrant activation of ERBBs in ligand-dependent and -independent ways in epithelial tumours promotes tumour cell proliferation, survival, migration and metastasis (Jorissen *et al*, 2003; Hynes and Lane, 2005). Recently, the role of EGF signalling in mediating tumour–stroma interaction in the tumour microenvironment becomes increasingly recognised (De Luca *et al*, 2008). In this review, we will first briefly overview the regulation and functions of EGF signalling pathway in mammalian development and tumourigenesis. Next, we will introduce the molecular mechanisms governing bone homoeostasis and bone metastasis. Finally, we will discuss several recent findings that established a role for EGF signalling in the complex network of tumour–stromal cross talks during the formation of osteolytic bone metastasis (Kim *et al*, 2003; Weber *et al*, 2003; Normanno *et al*, 2005; Lu *et al*, 2009). These studies provide the foundation for evaluating ERBBs and their ligands as potential therapeutic targets for controlling bone metastasis.

## The regulation and function of EGF signalling in development

The activity of the EGF signalling pathway is controlled at several levels. Under physiological conditions, the ERBB receptor activity is largely dependent on ligand availability, which is in part controlled post-translationally through a process called ectodomain shedding (Hynes and Lane, 2005; Wieduwilt and Moasser, 2008; Schneider and Wolf, 2009). The extracellular domain of EGF family ligands is cleaved by proteases belonging to families of matrix metalloproteinase (MMP), a disintegrin and metalloprotease (ADAM), and a disintegrin-like and metalloprotease with thrombospondin (ADAMTS). Ectodomain shedding occurs in response to diverse stimuli and most often after activation of G-protein-coupled receptors by ligands such as endothelin-1, bombesin, thrombin, lysophosphatidic acid and angiotensin-II (Wieduwilt and Moasser, 2008). Soluble EGF-like ligands can activate ERBBs on the same cell (autocrine), adjacent cells (paracrine) or cells in other organs (endocrine). The importance of ectodomain shedding in regulating ERBB activity is shown by the phenotypes from mice with endogenous HB-EGF replaced with either an uncleavable form or a constitutively soluble form of HB-EGF (Yamazaki *et al*, 2003). The former leads to severe heart failure and enlarged heart valves (similar to the phenotypes in HB-EGF-null and EGFR-null animals), whereas the latter causes hyperplasia in the skin and heart. Our recent study highlights the importance of ectodomain shedding of HB-EGF, AREG and TGF-*α* by proteases MMP1 and ADAMTS1 in the establishment of paracrine signalling cascade in bone metastasis (discussed below) (Lu *et al*, 2009). Besides the G-protein-coupled receptors, other signals can also activate protease-mediated ectodomain shedding, such as Wnt/Frizzled and oestradiol/oestrogen receptor (Hynes and Lane, 2005). Signal attenuation of EGF signalling is achieved through tyrosine dephosphorylation, receptor endocytosis and regulation of receptor dimerisation (Wieduwilt and Moasser, 2008). Systems biology studies reveal that the robustness and dynamic control of the EGF signalling is achieved by positive feedback loop (heterodimerisation with ERBB2, autocrine and paracrine), negative feedback loop (signal attenuation) and buffering (regulation of heterodimer formation and stability of ERBB2 by HSP90) (Citri and Yarden, 2006). Unfortunately, systems control in the signalling pathway can be overridden by pathological conditions, such as cancer, causing hyperactive signalling activity and uncontrolled cell proliferation.

The functions of ERBBs and EGF family ligands during mammalian development are studied by the gene targeting technique in mice. Deficiency in any of the four ERBBs leads to embryonic or perinatal death (Wieduwilt and Moasser, 2008). Phenotypic analyses of the knockout mice indicate that each ERBB receptor has indispensable roles in development and that functional redundancy among the receptors, if any, is minimal. In contrast, mice lacking genes encoding the EGF family ligands, including AREG, betacellulin, EGF, epiregulin and TGF-*α*, are viable and show much less severe phenotypes, indicating high level of redundancy. However, deficiency of the two EGF-like ligands, NRG1 and HB-EGF, does cause lethality, suggesting that in spite of the functional redundancy of EGF signalling in the development of some tissues, restricted ligand–receptor pairing is required for certain defined organs (Citri and Yarden, 2006; Wieduwilt and Moasser, 2008).

## EGF signalling in cancer and targeted therapeutics

The ERBB receptors and ligands are frequently overexpressed by carcinoma cells (Normanno *et al*, 2006). The EGFR and ERBB3 are overexpressed in 50–70% of lung, colon and breast carcinoma; ERBB2 is overexpressed in 30% of breast cancer patients; ERBB4 is expressed in 50% of breast cancer patients and 22% of colon cancer patients. Mutations of *ERBB* genes in cancer have been intensely investigated and found in all four members (Normanno *et al*, 2006; Wieduwilt and Moasser, 2008). For example, gene amplification and extracellular domain deletions of EGFR are found in 40–60% of glioblastoma multiforme patients. Kinase domain mutations of EGFR are found in 30–50% East Asian and 5–10% non-East Asian patients of non-small cell lung cancer. Gene amplification of ERBB2 is found in 20–30% of breast cancer patients. Gene amplification leads to gene overexpression, whereas extracelluar domain truncation and kinase domain mutations often render the receptors constitutively active. The ERBB2 amplification and overexpression in node-positive breast cancer patients are associated with poor prognosis independent of other markers (Ravdin and Chamness, 1995). Co-expression of two or more members of ERBB family is associated with a worse outcome (Normanno *et al*, 2006). Although mutation of EGF family ligands was not documented, the ligands are frequently expressed, and often co-expressed in breast, colon, lung, ovarian, gastric and prostate carcinomas (Normanno *et al*, 2006; De Luca *et al*, 2008). These results raise the possibility of constitutive activation of ERBB signalling by an autocrine loop.

The transforming activity of ERBB receptors and ligands are best demonstrated in mice with overexpression of these genes in the mouse mammary tissue. The EGFR overexpression leads to hyperplasia in virgins and dysplasia and tubular adenocarcinoma in lactating mice (Brandt *et al*, 2000). Overexpression of wild-type murine homologue of ERBB2 (*neu*) or activated *neu* leads to focal adenocarcinoma after long latency (Guy *et al*, 1992) or multifocal adenocarcinoma with a shorter latency (Muller *et al*, 1988), respectively. Transgenic overexpression of TGF-*α* causes a range of mammary abnormalities, including lobular hyperplasia, cystic hyperplasia, adenoma and adenocarcinoma (Matsui *et al*, 1990). In addition to stimulating tumour cell proliferation, EGF signalling has also been implicated in modulating functions of stromal cells in the tumour microenvironment, such as tumour-associated angiogenesis and bone metastasis (De Luca *et al*, 2008). Epidermal growth factor stimulates vascular endothelial growth factor expression in a panel of EGFR-positive cancer cells, including glioblastoma, gastric cancer, vulvar squamous carcinoma, bladder cancer and prostate cancer. The EGF family ligands can also exert direct mitogenic effects on tumour-associated endothelial cells (De Luca *et al*, 2008).

The EGF signalling network provides an array of therapeutic targets for the control of tumour growth. Most efforts have been focused on ERBB2 and EGFR. Currently, ERBB-targeted therapeutics in clinical use includes trastuzumab for metastatic breast cancer overexpressing ERBB2, cetuximab for colorectal cancer, gefetinib and erlotinib as a second-line treatment for non-small cell lung cancer (Hynes and Lane, 2005; Johnston *et al*, 2006). An emerging theme in the clinical application of ERBB inhibitors is the combined use with other drugs, such as chemotherapy, mammalian target of rapamycin inhibitor, anti-oestrogen agents or vascular endothelial growth factor receptor inhibitor (Hynes and Lane, 2005). Owing to the possibility of organ-specific functions of EGF signalling in promoting metastasis, clinical trials should measure both generalised and organ-specific outcome of metastasis.

## Mechanisms of bone homoeostasis and metastasis

Bone metastasis is the major cause of morbidity of several carcinomas. In one study involving 4399 patients, bone metastasis inflicts on 48% of breast cancer patients, 90% of prostate cancer patients, 39% of lung cancer patients and 35% of kidney cancer patients (Hess *et al*, 2006; Lu and Kang, 2007). It was estimated that 350 000 cancer patients die each year with bone metastasis (Mundy, 2002). Bone metastasis is often associated with bone pain, fracture, hypercalcemia, nerve compression syndromes, leukoerythroblastic anaemia and paralysis (Mundy, 2002; Roodman, 2004; Guise, 2009). Bone metastasis is classified as either osteolytic or osteoblastic, which actually represent two extremes of a continual spectrum of metastasis-associated bone pathology. Even though most bone metastases contain both osteolytic and osteoblastic components, metastases from breast and lung cancer usually are predominantly osteolytic, whereas metastases from prostate cancer are predominantly osteoblastic (Mundy, 2002). Bone lesion caused by myeloma is purely osteolytic.

Important insights of bone metastasis have been gained from a better molecular understanding of normal bone homoeostasis. The adult skeleton is constantly under coordinated remodelling that is mediated by a balanced activity of osteoblasts and osteoclasts (Mundy, 2002; Roodman, 2004; Guise, 2009). Osteoclast activation requires two cytokines, macrophage colony stimulating factor and receptor activator of nuclear factor-*κ*B ligand (RANKL), both of which are expressed by osteoblasts and stromal cells. The RANKL activity is antagonised by a soluble decoy receptor osteoprotegerin (OPG), which is also expressed by osteoblasts and stromal cells. Local RANKL/OPG ratio determines the osteoclastogenic potential. Binding of RANKL to RANK expressed on osteoclast precursors activates nuclear factor-*κ*B and Jun N-terminal kinase pathways to induce differentiation (Boyle *et al*, 2003). Therefore, most osteoclastogenic factors, including parathyroid hormone (PTH) and 1,25-dihydroxyvitamin D, stimulate osteoclast differentiation by indirectly signalling through osteoblasts and stromal cells to increase RANKL expression or to decrease OPG expression. Osteoblasts are derived from mesenchymal stem cells. Runx-2 is the essential transcription factor orchestrating the differentiation programme in osteoblast precursors (Harada and Rodan, 2003). Cytokines inducing osteoblast differentiation include bone morphogenetic proteins (BMPs), platelet-derived growth factor, fibroblast growth factors and TGF-*β*.

**Figure 1:**
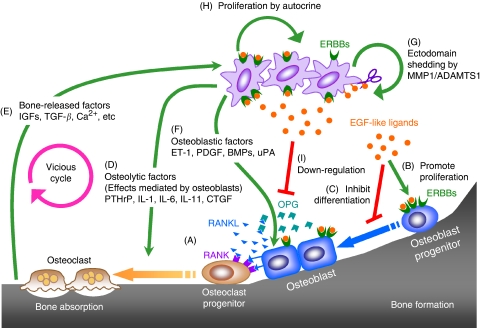
Multifunctional role of epidermal growth factor (EGF) signalling in bone development and metastasis. (A) In healthy bone, a proper level of osteoclast differentiation is ensured by a balance of receptor activator of nuclear factor-*κ*B ligand (RANKL) and osteoprotegerin (OPG) expression by osteoblasts and stromal cells. (B) During bone development, EGF-like ligands secreted by bone cells promotes the proliferation of osteoblast progenitors. (C) The EGF-like ligands secreted by bone cells inhibit differentiation of osteoblast progenitors. (D) Osteolytic tumour cells (e.g. breast cancer) express cytokines that regulate the expression of RANKL or OPG in osteoblasts, and ultimately activate osteoclasts and bone resorption. (E) Bone resorption releases growth factors and Ca^2+^ to promote tumour growth and more bone osteolysis. Continuous processes of D and E constitute the ‘vicious cycle’. (F) Osteoblastic tumour cells (e.g. prostate cancer) secret cytokines that activate osteoblast proliferation. (G) Overexpression of metalloproteinases, such as matrix metalloproteinase 1 (MMP1) and a disintegrin-like and metalloprotease with thrombospondin 1 (ADAMTS1), in metastatic tumour cells mediate ectodomain shedding of EGF-like ligands. (H) The soluble EGF-like ligands can promote tumour cell proliferation through an autocrine loop. (I) The soluble EGF-like ligands inhibit OPG expression from osteoblasts and tip the RANKL/OPG balance towards osteoclastogenesis. Whether EGF-like ligands from tumour cells can influence osteoblast progenitor proliferation (B) and differentiation (C) is unknown. CTGF, connective tissue growth factor.

In multiple myeloma, osteoclastogenic factors, such as interleukin-6, RANKL and macrophage inflammatory protein 1*α*, are abundantly expressed by myeloma cells (Roodman, 2004). Meanwhile, myeloma cells also express dickkopf 1, an inhibitor of osteoblast differentiation (Tian *et al*, 2003). These two mechanisms together can explain the purely osteolytic nature of bone lesions formed by myeloma. The osteolytic activity of breast cancer bone metastasis is partially because of the high expression level of PTH-related protein (PTHrP) in bone-metastatic tumour cells. The percentages of PTHrP-positive tumours in primary site, metastases in bone and metastases in other sites were 60, 92 and 17%, respectively (Powell *et al*, 1991). The PTHrP, together with other tumour-secreted factors, such as interleukin-1, interleukin-6 and interleukin-11, stimulates RANKL production or decreases OPG expression in osteoblasts and stromal cells. Elevated RANKL/OPG ratio favours osteoclast activation and bone resorption. Bone-released growth factors (e.g. insulin-like growth factor1 (IGF1), TGF-*β*) and calcium cause more PTHrP production and proliferation of tumour cells. The so-called ‘vicious cycle’ has the central role in the osteolysis and tumour growth of breast cancer metastasis to bone (Figure 1). Different set of molecules account for the osteoblastic nature of prostate metastasis to bone. Prostate cancer cells express osteoblastic factors, including endothelin-1, platelet-derived growth factor, TGF-*β*2, BMPs, urokinase plasminogen activator (uPA), fibroblast growth factors 1 and 2, which are all associated with proliferation of osteoblasts (Figure 1) (Mundy, 2002; Roodman, 2004). Osteoblastic metastases may be preceded by osteoclast activation and bone destruction, raising the possibility of targeting bone resorption in the treatment of bone metastasis of prostate cancer (Roodman, 2004). Currently, most drugs specifically targeting bone metastasis are inhibitors of bone resorption. Bisphosphanate blocks bone resorption by targeting osteoclasts and has been approved for the treatment of skeletal complications of malignancy. Bone metastasis drugs in clinical trials include OPG, RANK-Fc, PTHrP antibody and vitamin D analogues (Mundy, 2002). Clearly, identification of new molecules involved in osteolytic bone metastasis will provide new therapeutics possibilities.

## EGF signalling in bone development

The balance between osteoblasts and osteoclasts can be perturbed by altering the activity of EGF signalling. Early *in vitro* studies showed that addition of TGF-*α* and EGF to bone marrow culture, foetal rat long bone and neonatal mouse calvaria stimulated osteoclast formation and bone resorption (Ibbotson *et al*, 1986; Lorenzo *et al*, 1986; Takahashi *et al*, 1986). Phenotypes from genetically modified mice confirmed the important role of EGF signalling in bone formation. In both newborn EGFR-null mice (Wang *et al*, 2004) and 4-week-old AREG-null mice (Qin *et al*, 2005), trabecular bone area was decreased compared with wild-type mice, indicating the imbalance of bone remodelling. The EGFR-deficiency causes delayed primary endochondral ossification during embryonic development due to impaired osteoclast recruitment (Wang *et al*, 2004). Genetic evidence also suggested the involvement of EGFR in the regulation of osteoblasts. In hEGFR^KI^ knock-in mice, in which the human *EGFR* cDNA was used to replace the mouse *Egfr* gene, hypomorphic expression of *hEGFR* in the knock-in animals reduces the proliferation and increases the differentiation of primary calvarial osteoblasts (Sibilia *et al*, 2003). In EGF transgenic mice, abnormal accumulation of osteoblasts in the periosteum and endosteum was observed (Chan and Wong, 2000). Furthermore, growth retardation was observed in hEGFR^KI/KI^ mice (Sibilia *et al*, 2003) and EGF/AREG/TGF-*α* triple-knockout mice (Kelly *et al*, 2001), possibly due to impaired longitudinal growth of long bones (Sibilia *et al*, 2003). Curiously, slower growth was also observed in EGF transgenic mice, possibly through a different mechanism involving reduced level of serum insulin-like growth factor-binding protein-3 (Chan and Wong, 2000). The EGFR is shown to be expressed in osteoblasts but not in osteoclasts (Zhu *et al*, 2007). Taking the mouse phenotype and expression data together, the main function of EGF signalling in balancing osteoblast/osteoclast activities seems to stimulate the proliferation of preosteoblasts while inhibiting their full differentiation (Figure 1). By doing this, EGF signalling can indirectly influence osteoclast number and activity. The indirect role of EGF signalling in osteoclastogenesis was explored using either the mesenchymal stem cell-like cells (Normanno *et al*, 2005) or osteoblast cell lines (Zhu *et al*, 2007; Lu *et al*, 2009), and the results suggest that activation of the signalling pathway upregulates RANKL, macrophage colony stimulating factor and MCP-1, but downregulates OPG (Figure 1). The overall consequence of these regulations is enhanced osteoclast differentiation. This notion has direct implications in osteolytic bone metastasis growth, because EGF family ligands are frequently expressed in carcinomas (as discussed above). It is conceivable that by interacting with osteoblasts through paracrine EGF signalling, the tumour cells can ultimately promote osteoclastogenesis. Indeed, this possibility was confirmed in our recent study (Lu *et al*, 2009).

## EGF signalling in osteolytic bone metastasis

Involvement of EGF signalling in the pathogenesis of bone metastasis was implicated by the unexpected relief of bone pain in phase II clinical trials of EGFR inhibitor gefitinib in breast cancer patients (Albain *et al*, 2002; von Minckwitz *et al*, 2005). The inhibitory effect on tumour growth in bone can be exerted through two possible mechanisms: by directly blocking tumour cell proliferation, or by indirectly blocking tumour–stroma signalling cross talks that are essential for the continuous metastasis development. Results from a number of studies that evaluated the therapeutic benefit of targeting EGF signalling in preclinical bone metastasis models suggest that both mechanisms may be involved, and sometimes simultaneously. Bone metastasis formed by ERBB2-overexpressing breast cancer cell, BT474, was significantly blocked with trastuzumab, presumably due to reduced MARK pathway activity in tumour cells (Khalili *et al*, 2005). Blocking EGFR phosphorylation with the tyrosine kinase inhibitor, PKI166, reduced bone metastasis formation and bone destruction in a renal cell carcinoma model (Weber *et al*, 2003). Interestingly, both tumour cells and EGFR-expressing endothelial cells were induced to undergo apoptosis by the agent, suggesting that inhibition of angiogenesis may contribute to the efficacy of the drug. The inhibitory effect of PKI166 on prostate cancer bone metastasis has also been attributed to the targeting of both tumour cells and endothelial cells (Kim *et al*, 2003). In another study on prostate cancer bone metastasis, gefitinib inhibited the growth of prostate cancer cell PC3 and its highly bone-metastatic subline PCb2 at the similar level when cells were grown as subcutaneous tumours, yet osteolytic bone metastasis was inhibited to a larger extent in PCb2 (Angelucci *et al*, 2006). The differential effect on bone metastasis cannot be readily explained by direct targeting, as EGFR expression was comparable in two cell lines. Compared with the parental cell, PCb2 expressed higher level of uPA and conditioned medium induced more RANKL expression in the osteoblasts. The enhancement of RANKL expression was blocked by gefitinib treatment, thus linking the tumour-inhibitory effect of EGFR inhibitors to RANKL-mediated osteoclast activation.

In one of our recent studies, we showed direct evidence for reducing bone metastasis growth through inhibiting EGF signalling in bone stromal cells (Lu *et al*, 2009). In a bone metastasis model of breast cancer, bone-metastatic variants of the MDA-MB-231 breast cancer cell line were found to overexpress two metalloproteinases, MMP1 and ADAMTS1. Ectodomain shedding by the proteases releases three EGF-like factors, HB-EGF, AREG and TGF-*α*, from tumour cells. The soluble factors activates EGFR pathway in adjacent osteoblasts through a paracrine mechanism and downregulates OPG expression. The increased ratio of RANKL and OPG favours osteoclastogenesis and contributes to the vicious cycle of osteolytic bone metastasis (Figure 1). The EGFR inhibitor-insensitive MDA-MB-231 cells offered the opportunity to test the indirect effect of targeting EGFR in bone metastasis. Cetuximab and gefitinib markedly impeded bone metastasis formation when the highly bone-metastatic subline of MDA-MB-231 was inoculated into mice by intracardiac or intratibia injection. Combining two drugs produced even more significant inhibition. Together, these results provide a rationale for targeting EGF-like factors (HB-EGF, AREG and TGF-*α*) and proteases (MMP1 and ADAMTS1) in tumour cells, as well as EGFR in osteoblasts for controlling osteolytic bone metastasis of breast cancer.

## Conclusion and future direction

The chance of long-term survival for patients with bone metastasis is usually dismal. For example, only 20% of breast cancer patients live more than 5 years after the diagnosis of bone metastasis (Roodman, 2004). Therefore, finding effective therapeutic targets is urgently needed for controlling this debilitating disease. Studies of EGF signalling functions in bone development and metastasis suggest EGF signalling pathway as a promising new target for controlling osteolytic bone metastasis. It is unknown whether osteoblastic metastasis can also be reduced by ERBB inhibitors. To further determine the efficacy and general applicability of the strategy, testing the therapeutic benefit of EGF signalling inhibition in additional preclinical models of both osteolytic and osteoblastic bone metastasis should be performed. One cancer type that has not been tested but may benefit from ERBB-targeting therapeutics is myeloma. Both ERBB receptor and ligands are well documented to be expressed by myeloma cells (Johnston *et al*, 2006). Finally, clinical trials of using ERBB inhibitors to specifically reduce bone metastasis and the associated morbidities are needed to translate recent exciting findings into more effective treatments for cancer patients with bone metastases.
